# Enhanced recovery after surgery in patients undergoing scoliosis correction surgery: protocol for a systematic review and meta-analysis

**DOI:** 10.3389/fmed.2026.1872301

**Published:** 2026-07-28

**Authors:** Jixin Chen, Qinxin Zhou, Huahai Yao, Jiafeng Zhang, Yong Xu, Jianliang Chen, Ci Wang, Feng Ye

**Affiliations:** 1 Department of Orthopaedic Surgery, Shaoxing Shangyu District Hospital of Traditional Chinese Medicine, Shaoxing, China; 2 Department of Orthopaedic Surgery, Shaoxing Hospital of Traditional Chinese Medicine Affiliated to Zhejiang Chinese Medical University, Shaoxing, China; 3 The First Affiliated Hospital of Zhejiang Chinese Medical University (Zhejiang Provincial Hospital of Chinese Medicine), Hangzhou, China

**Keywords:** enhanced recovery after surgery, ERAS, meta-analysis, perioperative care, scoliosis, spinal deformity, systematic review

## Abstract

**Background:**

Scoliosis correction surgery is associated with significant perioperative morbidity and prolonged recovery. Enhanced Recovery After Surgery (ERAS) protocols, which employ evidence-based, multimodal perioperative care, have been increasingly used in spinal surgery to improve patient outcomes. However, their efficacy and safety in scoliosis correction surgery remain unclear.

**Methods:**

This protocol describes a systematic review and meta-analysis following PRISMA-P guidelines. We will systematically search electronic databases from inception to January 2026 for randomized controlled trials and observational studies comparing ERAS protocols with conventional care in patients undergoing scoliosis correction surgery. The primary outcome will be length of hospital stay. Secondary outcomes will include postoperative complications, pain intensity, opioid consumption, time to ambulation, readmission rates, patient satisfaction, healthcare costs, and adverse events. Two independent reviewers will screen studies, extract data, and assess methodological quality using the Cochrane Risk of Bias tool version 2 for randomized trials and the Risk of Bias in Non-randomized Studies - of Interventions tool for non-randomized studies. Meta-analysis will be performed using Review Manager version 5.4 and Stata version 17 when feasible. The certainty of evidence will be assessed using the GRADE approach.

**Discussion:**

This systematic review and meta-analysis will synthesize current evidence on the effectiveness and safety of ERAS protocols in scoliosis correction surgery. The findings are expected to inform clinical practice, support guideline development, and highlight gaps for future research. Results will be disseminated via peer-reviewed publication and scientific meetings.

**Systematic review registration:**

PROSPERO, CRD420251089411

## Introduction

Scoliosis is a three-dimensional spinal deformity characterized by lateral curvature and vertebral rotation that affects approximately 2–3% of the population, predominantly children, adolescents, and adults. Surgical correction, typically involving posterior spinal fusion with instrumentation, is often indicated in cases of progressive or severe curvature, generally when the Cobb angle exceeds 40–50 degrees, to prevent functional deterioration, reduce pain, and improve quality of life (1). However, scoliosis correction surgery represents a major orthopedic procedure associated with significant perioperative morbidity, including substantial blood loss often ranging from 500 to 2000 mL, intense postoperative pain, prolonged immobilization, and increased risk of complications such as surgical site infection, pulmonary dysfunction, neurological deficits, and thromboembolic events (2, 3). These challenges frequently result in extended hospital stays typically lasting 5–10 days, high healthcare costs, and delayed return to normal activity.

Enhanced Recovery After Surgery represents a multidisciplinary, evidence-based approach designed to reduce the physiological and psychological stress of surgery, accelerate recovery, and improve clinical outcomes while maintaining or improving patient safety (4). Originally developed by Henrik Kehlet for colorectal surgery in the 1990s, ERAS protocols integrate various perioperative elements into a coordinated, evidence-based care pathway (5). These elements span the entire perioperative period and include preoperative patient education and counseling, optimization of nutritional status, multimodal analgesia, opioid-sparing strategies, goal-directed fluid therapy, prevention of hypothermia, early removal of urinary catheters, early mobilization, and early oral nutrition (6). In recent years, the principles of ERAS have been increasingly adapted for use in orthopedic and spinal surgeries, with growing evidence suggesting potential benefits in reducing length of hospital stay, perioperative complications, and opioid consumption (7). Several studies in adult spinal surgery populations have demonstrated that ERAS protocols may reduce hospital length of stay by one to three days, decrease postoperative complications by 20–40%, and reduce opioid requirements by 30–50% compared to conventional care (8, 9). Specifically for adolescent idiopathic scoliosis, recent meta-analyses have shown significant reductions in length of stay without increasing complication or readmission rates (10, 11). However, the implementation and evaluation of ERAS protocols specifically for scoliosis correction surgery remain limited and inconsistent.

Despite this progress, several important knowledge gaps persist. Existing studies are often heterogeneous in design, with protocol elements ranging from three to fifteen different interventions, and outcome measures that are sometimes conflicting or inconclusive (12, 13). There is no widely accepted standardized ERAS pathway tailored to the unique needs of patients undergoing scoliosis surgery, who often require multidisciplinary management involving orthopedic surgeons, anesthesiologists, pain specialists, physiotherapists, and nursing staff. Most available evidence comes from heterogeneous spinal surgery populations or procedures distinct from scoliosis correction, such as lumbar fusion or cervical surgery, limiting the generalizability of these findings to scoliosis patients who typically undergo longer, more extensive procedures with greater blood loss and pain. Furthermore, the pediatric and adolescent population represents a substantial proportion of scoliosis surgery patients and has unique physiological characteristics, pain management needs, and recovery expectations that may differ from adult surgical populations (14, 15). The applicability of ERAS principles to this younger population requires specific evaluation. Multimodal analgesia protocols, including the use of regional anesthesia techniques, intrathecal morphine, and opioid-sparing medications such as gabapentinoids and ketamine, have shown promise in reducing postoperative pain and opioid consumption in this population (16, 17). Additionally, while some individual ERAS components have been studied in isolation, the synergistic effect of implementing multiple evidence-based interventions as part of a coordinated pathway remains incompletely understood in the context of scoliosis surgery (18).

Given the increasing adoption of ERAS concepts in spine surgery and the clinical importance of optimizing perioperative care for scoliosis patients, there is a clear need to systematically review and synthesize the current evidence (19, 20). This systematic review and meta-analysis aims to evaluate the effectiveness of ERAS protocols compared to conventional care in reducing length of hospital stay in patients undergoing scoliosis correction surgery as the primary objective. Secondary objectives include assessing the impact of ERAS protocols on postoperative complications, pain intensity, opioid consumption, time to ambulation, readmission rates, patient satisfaction, and healthcare costs; evaluating the safety profile of ERAS protocols by examining adverse events; exploring potential effect modifiers through pre-specified subgroup analyses; and assessing the certainty of evidence using the GRADE approach. A rigorous evaluation of the effectiveness and safety of ERAS protocols in this population will help clarify their impact on clinical outcomes, inform best practices, and guide future research aimed at establishing standardized, evidence-based perioperative care pathways for scoliosis correction surgery.

### Comparison with published systematic reviews

Several recent systematic reviews and meta-analyses have evaluated ERAS protocols in spinal surgery and scoliosis populations, though with notable differences in scope, population, and methodology. To clarify the incremental contribution of the present review, we systematically compared our protocol with six key published meta-analyses (5, 9–13) across the following dimensions: target population, scoliosis etiology coverage, age range, search cutoff date, ERAS operational definition, risk of bias assessment tool, and use of GRADE evidence certainty grading. The present review offers four core incremental values beyond existing syntheses. First, broader population coverage: most prior reviews focus exclusively on adolescent idiopathic scoliosis (AIS), whereas this review includes both adolescent and adult patients across all etiologies (idiopathic, congenital, neuromuscular, degenerative), filling the evidence gap for adult deformity and non-idiopathic scoliosis populations. Second, updated evidence base: our search extends to January 2026, incorporating all studies published after 2024 (the typical cutoff of most existing meta-analyses), thereby capturing the latest real-world evidence from ERAS implementation. Third, methodological rigor: we apply the most current bias assessment tools (RoB 2.0 for RCTs, ROBINS-I for non-randomized studies) and standard GRADE evidence certainty grading, which have been inconsistently used in prior scoliosis ERAS reviews. Fourth, comprehensive outcome domains: we systematically evaluate safety profiles, healthcare costs, equity characteristics (via the PROGRESS-Plus framework), and component-level effects, which are rarely reported or synthesized in existing reviews. Existing reviews also have notable methodological limitations that this review aims to address, including high clinical heterogeneity from mixing diverse surgical populations and ERAS protocols, inconsistent or absent ERAS operational definitions, lack of stratified analyses by etiology and age group, and limited assessment of evidence certainty. By explicitly defining the scope, population, and analytical framework *a priori*, this protocol provides a transparent and comprehensive plan to address these gaps.

## Methods

### Protocol and registration

This systematic review protocol is developed in accordance with the Preferred Reporting Items for Systematic Review and Meta-Analysis Protocols guidelines and has been registered in the International Prospective Register of Systematic Reviews with registration number CRD420251089411. Any amendments to this protocol will be documented and reported in the final manuscript with appropriate justification. The completed PRISMA-P checklist is provided in Supplementary file 1.

### Eligibility criteria

We will use the PICOS framework to define our eligibility criteria, encompassing Population, Intervention, Comparator, Outcomes, and Study design. For the purposes of this review, an ERAS protocol is defined as a structured, multimodal perioperative care pathway incorporating a minimum of three evidence-based components spanning at least two perioperative phases (preoperative, intraoperative, or postoperative). Studies implementing only a single intervention (e.g., a standalone analgesic protocol) without integration into a broader perioperative care bundle will be excluded. A structured table of ERAS components (Supplementary file 2). This threshold is aligned with the core principle of ERAS as a multimodal, integrated perioperative care pathway rather than a single intervention, consistent with the ERAS Society’s conceptual framework for spinal surgery. It is also consistent with the operational definition used in the majority of published ERAS systematic reviews in spinal deformity surgery (10–12), ensuring comparability with existing evidence. The requirement of spanning at least two perioperative phases (preoperative, intraoperative, postoperative) ensures the intervention reflects a full-pathway optimization rather than single-stage improvement, which is the defining feature of ERAS.

### Population

Eligible populations will include adolescent and adult patients aged 10 years or older undergoing scoliosis correction surgery, regardless of etiology including idiopathic, congenital, neuromuscular, or degenerative scoliosis. We will exclude patients undergoing spinal surgery for conditions other than scoliosis, such as isolated kyphosis, spondylolisthesis, spinal stenosis, disc herniation, tumor, infection, or trauma. Studies focusing on revision scoliosis surgery, non-surgical interventions, or pediatric patients under 10 years of age will be excluded due to substantially different physiological characteristics and care requirements.

### Intervention

The intervention of interest is the implementation of an ERAS protocol during the perioperative period of scoliosis correction surgery. For the purposes of this review, an ERAS protocol will be defined as a structured, multimodal perioperative care pathway that includes at least three evidence-based elements spanning the preoperative, intraoperative, and postoperative periods. Preoperative elements may include patient education and counseling, nutritional assessment and optimization, avoidance of prolonged fasting with allowance of clear fluids up to two hours before surgery, avoidance of routine premedication with sedatives, and preoperative carbohydrate loading. Intraoperative elements may comprise standardized anesthetic protocols, multimodal analgesia using combinations of non-opioid analgesics, opioid-sparing strategies, goal-directed fluid therapy, maintenance of normothermia, blood conservation strategies such as tranexamic acid administration or cell salvage, and minimally invasive or tissue-sparing surgical techniques where applicable. Postoperative elements may include multimodal postoperative analgesia, early removal of urinary catheters within 24 h, early mobilization protocols with sitting on postoperative day zero or one and walking within 48 h, early oral nutrition with clear liquids within 4–6 h and solid food as tolerated, prevention of postoperative nausea and vomiting, structured discharge criteria, and post-discharge follow-up plans. Studies must clearly describe their ERAS protocol and specify which elements were implemented. If the number or nature of ERAS elements is unclear from the publication, we will contact the corresponding authors for clarification.

### Comparator

The comparator will be standard or conventional perioperative care that does not involve a structured ERAS pathway. Conventional care will be defined as routine perioperative management according to the institution’s standard protocols, which may include some individual elements of ERAS but not as part of a coordinated, multimodal pathway.

### Outcomes

The primary outcome will be length of hospital stay, defined as the number of days from surgery completion to hospital discharge, reported as mean with standard deviation or median with interquartile range.

Secondary outcomes will encompass multiple domains. Postoperative complications will be assessed within 30 days and will include overall complication rates as well as specific complications categorized into surgical complications such as infection, wound dehiscence, hardware failure, dural tear, and neurological deficit, and medical complications including pulmonary complications, urinary tract infection, thromboembolic events, and cardiac events. Complications will also be classified according to severity using the Clavien-Dindo grading system, distinguishing between minor complications of grade I-II and major complications of grade III-V.

Pain intensity will be measured using validated pain scales including the Visual Analog Scale, Numeric Rating Scale, or equivalent instruments, assessed at 24 h, 48 h, 72 h postoperatively, at hospital discharge, and at two weeks postoperatively when available.

Opioid consumption will be quantified as total cumulative opioid use converted to oral morphine equivalent dose in milligrams during the first 24 h, 48 h, 72 h postoperatively, throughout the total hospital stay, and during the first two weeks post-discharge when data are available.

Additional secondary outcomes include time to first ambulation defined as hours from end of surgery to when the patient first sits upright or stands or walks; unplanned hospital readmission within 30 days of discharge; patient satisfaction measured using validated instruments such as visual analog scales for satisfaction or the Patient Satisfaction Questionnaire; healthcare costs encompassing operating room costs, hospital stay costs, medication costs, and total episode costs reported in original currency and converted to US dollars when possible for comparison purposes; adverse events directly attributable to ERAS protocol implementation including any safety concerns, protocol non-compliance, or harm specifically related to ERAS interventions; quality of life measured using validated instruments such as EQ-5D, SF-36, or SRS-22 at six weeks, three months, or six months postoperatively when available; and time to return to normal activities including return to school or work measured in days.

We will extract outcomes at all available time points but will prioritize outcomes assessed at 30 days postoperatively for primary analysis. The minimum follow-up duration required for study inclusion will be until hospital discharge.

### Study design

Eligible study designs will include randomized controlled trials, quasi-experimental studies defined as non-randomized controlled trials with concurrent controls, prospective cohort studies with concurrent controls, and retrospective cohort studies with concurrent controls provided that adjusted analysis controlling for key confounders is performed. We will exclude case reports and case series involving fewer than 10 patients per group, cross-sectional studies, studies without a comparator group, conference abstracts without full-text publication unless authors can provide complete data, editorials, commentaries, letters, reviews whether systematic or narrative, protocols without results, and animal studies. There will be no restrictions on publication date, language, or geographic location. Studies in languages other than English will be translated using professional translation services or native speakers within the research team where available. Both published and unpublished studies including grey literature will be eligible if they meet other inclusion criteria.

## Information sources and search strategy

### Databases and sources

A comprehensive literature search will be conducted from database inception to January 2026, in multiple electronic databases. Primary databases will include PubMed/MEDLINE via the PubMed interface, Embase via the Ovid platform, the Cochrane Central Register of Controlled Trials, and Web of Science Core Collection. Additional databases to be searched include CNKI for Chinese language literature, Scopus, and CINAHL. We will employ ASReview (version ≥1.0), an open-source active learning software, to assist with title and abstract screening. ASReview will be used in the following manner: (1) an initial training set of 50 records (25 relevant, 25 irrelevant) will be manually labeled by two independent reviewers; (2) the algorithm will subsequently prioritize records predicted to be relevant; (3) screening will continue until a stopping criterion of 95% recall is achieved (i.e., when the model predicts that ≥95% of all relevant records have been identified). All final inclusion/exclusion decisions will be verified manually by two independent reviewers, with discrepancies resolved by consensus. To minimize publication bias, we will contact a minimum of ten leading researchers and clinical experts in the field of ERAS and spinal surgery. Contact will be made via email to corresponding authors of the most-cited relevant studies and to members of the ERAS Society Spine Surgery Working Group. Experts will be asked to share unpublished datasets, conference abstracts, and ongoing trial registrations. We will also search ClinicalTrials.gov, the WHO ICTRP, and grey literature databases including OpenGrey and ProQuest Dissertations and Theses Global. Reference lists of all included studies and relevant systematic reviews will be manually screened for additional eligible studies. Forward citation tracking of included studies will be performed using Web of Science and Google Scholar. We will also contact experts in the field and relevant professional societies, including the Scoliosis Research Society and the ERAS Society, to identify unpublished or ongoing studies that may meet our eligibility criteria.

### Search strategy development

The search strategy will be developed by a medical librarian with expertise in systematic reviews in collaboration with the research team. We will use a combination of Medical Subject Headings and free-text keywords related to the population, intervention, and study design. The search will be sensitive rather than specific to ensure comprehensive capture of relevant literature. The search strategy will be peer-reviewed by a second librarian using the Peer Review of Electronic Search Strategies checklist before implementation. The core search concepts will include terms related to scoliosis and spinal deformity, terms related to enhanced recovery after surgery and perioperative care, and terms for relevant study designs. For the scoliosis concept, we will use terms such as scoliosis, spinal curvature, spinal deformity, spine deformity, and kyphoscoliosis. For the ERAS concept, terms will include enhanced recovery, ERAS, fast-track, rapid recovery, accelerated rehabilitation, perioperative care, perioperative pathway, multimodal care, and related variants. Study design filters will be applied where appropriate to capture randomized controlled trials, controlled clinical trials, cohort studies, and comparative studies. The full search strategy for PubMed is provided, and will be adapted for other databases accounting for differences in subject headings, syntax, and search interfaces. We will not use language restrictions in our searches, though we will document the languages of included studies. The searches will be re-run immediately before final analysis to capture any newly published studies, and we will retrieve any additional eligible studies for inclusion.

An example of the PubMed search strategy includes the following structure: combining population terms such as “Scoliosis”[Mesh] OR scoliosis[tiab] OR “spinal curvature”[tiab] OR “spinal deformity”[tiab] OR “spine deformity”[tiab], with intervention terms such as “enhanced recovery”[tiab] OR ERAS[tiab] OR “fast track”[tiab] OR “rapid recovery”[tiab] OR “accelerated rehabilitation”[tiab] OR “perioperative care”[Mesh] OR “perioperative pathway”[tiab] OR “multimodal care”[tiab], and study design filters for controlled trials and comparative studies. Complete search strategies for all databases will be provided as Supplementary materials with the published systematic review.

### Study selection

All citations retrieved from the searches will be imported into EndNote version 20 for initial duplicate removal, followed by import into Rayyan, a web-based systematic review management software, for screening. Two reviewers will independently screen titles and abstracts of all retrieved records against the eligibility criteria. Prior to formal screening, calibration exercises will be conducted on a random sample of 50 records to ensure consistency and clarify any ambiguities in the eligibility criteria. During calibration, reviewers will discuss discrepancies and refine their interpretation of the inclusion and exclusion criteria until adequate agreement is achieved. Following title and abstract screening, full-text articles of potentially eligible studies will be retrieved and assessed independently by the same two reviewers against the detailed inclusion criteria. For studies where eligibility is unclear, the full text will be obtained for further evaluation. Any disagreements between reviewers at either screening stage will be resolved through discussion to reach consensus. If consensus cannot be achieved, a third senior reviewer will adjudicate. We will calculate inter-rater agreement for both title/abstract and full-text screening using Cohen’s kappa statistic. Kappa values will be interpreted as poor agreement when *κ* ≤ 0.2, fair agreement when 0.21 ≤ κ ≤ 0.4, moderate agreement when 0.41 ≤ κ ≤ 0.6, substantial agreement when 0.61 ≤ κ ≤ 0.8, and almost perfect agreement when κ > 0.8. If kappa values fall below 0.6 at either screening stage, we will conduct additional calibration exercises and consider retraining or substituting reviewers to improve consistency.

Reasons for exclusion at the full-text stage will be documented in detail. The entire study selection process will be illustrated using a PRISMA flow diagram showing the number of records identified through database searching and other sources, the number of records after duplicates removed, the number of records screened, the number of full-text articles assessed for eligibility, and the final number of studies included in qualitative synthesis and quantitative synthesis, along with reasons for exclusions at each stage. For studies published in languages other than English, we will use translation services or consult with native speakers within our extended research network. If full translation is not feasible, we will translate the abstract and methods section to determine eligibility, and if eligible, will obtain full translation for data extraction. For ongoing studies identified through trial registries, we will contact the principal investigators to inquire about completion status and availability of results.

### Data extraction

Data extraction will be performed independently by two trained reviewers using a standardized, pre-piloted data extraction form developed specifically for this review in Microsoft Excel. Prior to formal data extraction, the form will be piloted on three to five included studies to ensure it captures all relevant information and that reviewers interpret data fields consistently. Any necessary modifications to the form will be made based on the pilot exercise. The data extraction form will capture comprehensive information across multiple domains. Study characteristics to be extracted include first author surname, publication year, country of study conduct, study design, study setting including hospital type and surgical volume, sample size calculation and whether the study was adequately powered, funding sources, and any reported conflicts of interest. Population characteristics will include the number of participants in intervention and control groups, age range or mean age with standard deviation, sex distribution, body mass index when reported, scoliosis etiology including proportion with idiopathic, congenital, neuromuscular, or degenerative scoliosis, preoperative Cobb angle mean with standard deviation or median with interquartile range, levels of fusion, preoperative comorbidities when specified, and any reported baseline differences between groups. Surgical characteristics to be documented include specific surgical procedure performed such as posterior spinal fusion with or without anterior release, surgical approach whether posterior only or combined anterior–posterior, use of instrumentation type, estimated blood loss, operative duration, intraoperative complications, and any intraoperative deviations from protocol. Intervention details will be comprehensively extracted, documenting the complete ERAS protocol as implemented including the total number of ERAS components, specific listing of each component across preoperative, intraoperative, and postoperative phases, details of each component such as timing, dosing for medications, and specific protocols for mobilization, protocol adherence rates when reported, any co-interventions, modifications made to the protocol during the study, and training provided to staff for protocol implementation.

Control group details will include description of standard care practices, which specific elements if any were common between ERAS and control groups, historical practices that may have changed during the study period, and any co-interventions in the control group. For each outcome, we will extract the specific outcome measure or instrument used, timing of assessment, number of participants analyzed for each outcome, results for intervention and control groups reported as means with standard deviations for continuous outcomes or proportions with numerators and denominators for dichotomous outcomes, measures of effect with confidence intervals when provided, *p*-values, and any adjusted analyses including which covariates were adjusted for. Additional information to be extracted includes duration of follow-up, loss to follow-up and reasons, any protocol violations, adverse events related to ERAS or the surgical procedure, subgroup analyses performed by study authors, and key conclusions stated by the authors. When data are missing, unclear, or only presented graphically, we will attempt to contact the corresponding authors by email up to three times over a six-week period. All correspondence will be documented including dates of contact attempts and responses received. For graphical data, we will use digital ruler software or plot digitizer tools to extract numerical values when author contact is unsuccessful. We will conduct sensitivity analyses excluding studies with imputed or extracted data to assess the impact on results. For studies with multiple publications reporting on the same patient cohort, we will link all reports and extract data from the primary publication, supplementing with additional details from secondary publications where available, ensuring that participants are not double-counted in our analyses. Any disagreements in data extraction will be resolved through discussion between the two reviewers, and when consensus cannot be reached, a third senior reviewer will be consulted. We will maintain detailed records of all decisions and clarifications made during data extraction.

### Risk of bias assessment

The methodological quality and risk of bias of included studies will be assessed independently by two trained reviewers using instruments appropriate to study design. Prior to formal assessment, calibration exercises will be conducted on a sample of included studies to ensure consistent application of the tools. For randomized controlled trials, we will use the Cochrane Risk of Bias tool version 2. This tool assesses five domains: bias arising from the randomization process, bias due to deviations from intended interventions, bias due to missing outcome data, bias in measurement of the outcome, and bias in selection of the reported result. For each domain, we will answer signaling questions that inform an algorithm-based judgment of low risk of bias, some concerns, or high risk of bias. An overall risk of bias judgment will be made for each outcome within each study. Studies will be judged as having low overall risk of bias if all domains are rated as low risk, some concerns if at least one domain raises some concerns but no domain is at high risk, or high overall risk of bias if at least one domain is at high risk or multiple domains raise some concerns in ways that substantially lower confidence in the result. Specific considerations for the RoB 2 assessment in our context include that blinding of participants and personnel will be difficult to achieve for ERAS protocols given their multimodal nature, but we will assess whether lack of blinding could have affected the outcomes; blinding of outcome assessors will be particularly important for subjective outcomes such as pain and satisfaction; missing outcome data will be carefully evaluated given potential for differential dropout related to intervention tolerability; and we will check for selective reporting by comparing protocols when available with published results and examining whether all expected outcomes are reported. For non-randomized studies including quasi-experimental and cohort studies, we will apply the ROBINS-I tool, which evaluates seven domains: bias due to confounding, bias in selection of participants into the study, bias in classification of interventions, bias due to deviations from intended interventions, bias due to missing data, bias in measurement of outcomes, and bias in selection of the reported result. For each domain, signaling questions guide judgment of low risk, moderate risk, serious risk, or critical risk of bias, with an option for no information when insufficient detail is provided. An overall risk of bias judgment will be made, with studies classified as low risk if all domains are at low risk, moderate risk if all domains are at low or moderate risk, serious risk if at least one domain is at serious risk, or critical risk if at least one domain is at critical risk. Key confounders that should be controlled for in observational studies of ERAS protocols include patient age, sex, body mass index, scoliosis etiology, severity of scoliosis as measured by Cobb angle, number of levels fused, comorbidities, baseline pain levels, and surgical approach. Studies will be assessed for whether these confounders were measured and controlled through matching, stratification, multivariable adjustment, or other methods. Studies that fail to control for key confounders will be judged at serious or critical risk of bias due to confounding. Risk of bias assessments will be performed at the outcome level, recognizing that risk may vary between outcomes within the same study, particularly for subjective versus objective outcomes. Results of risk of bias assessments will be presented in tables and figures, including summary plots showing the proportion of studies with each risk of bias judgment across domains. Any disagreements in risk of bias assessment will be resolved through discussion between reviewers, and when necessary, consultation with a third senior reviewer. We will maintain transparent documentation of judgments and their supporting rationale. These assessments will inform our GRADE evaluations and sensitivity analyses.

## Data synthesis and statistical analysis

### Measures of treatment effect

For continuous outcomes including length of hospital stay, pain scores, opioid consumption, time to ambulation, and healthcare costs, we will calculate mean differences with 95% confidence intervals. When different instruments or scales are used to measure the same construct, such as different pain scales, we will calculate standardized mean differences to enable pooling across studies. For interpretation, standardized mean differences of 0.2 will be considered small, 0.5 moderate, and 0.8 large effects according to Cohen’s conventions. For dichotomous outcomes including complication rates, readmission rates, and adverse events, we will calculate risk ratios with 95% confidence intervals. We will choose risk ratios over odds ratios due to their more intuitive interpretation, particularly when event rates are not rare. However, if included studies predominantly report odds ratios and conversion to risk ratios is not feasible, we will use odds ratios. All analyses will be two-tailed with statistical significance defined as *p* < 0.05. Effect estimates will be accompanied by 95% confidence intervals, and we will focus interpretation on both statistical significance and clinical importance of effect sizes.

### Assessment of heterogeneity

Statistical heterogeneity across studies will be assessed using multiple complementary methods. We will visually inspect forest plots for overlap of confidence intervals and consistency in the direction and magnitude of effects. Quantitatively, heterogeneity will be assessed using the Chi-squared test, with *p* < 0.10 indicating significant heterogeneity given the low power of this test, and the I^2^ statistic, which describes the percentage of variability in effect estimates that is due to heterogeneity rather than sampling error. We will interpret I^2^ values according to the following thresholds: I^2^ values of 0–40% may not be important heterogeneity, 30–60% may represent moderate heterogeneity, 50–90% may represent substantial heterogeneity, and 75–100% represents considerable heterogeneity, recognizing that these thresholds are not absolute and interpretation should also consider the magnitude and direction of effects and strength of evidence for heterogeneity from the Chi-squared test. When substantial heterogeneity is identified, we will explore potential sources through pre-specified subgroup analyses and meta-regression. If heterogeneity remains very high and cannot be adequately explained, we may determine that meta-analysis is not appropriate and will provide a narrative synthesis instead.

### Meta-analysis

When studies are sufficiently homogeneous in terms of population, intervention, comparator, and outcomes, we will conduct meta-analyses using Review Manager version 5.4 software. We will use random-effects models for all meta-analyses using the DerSimonian and Laird method, which assumes that true effects vary across studies due to differences in populations, interventions, and study conduct. Random-effects models provide a more conservative estimate and are generally more appropriate when pooling evidence from different settings and populations. As sensitivity analyses, we will also conduct fixed-effect meta-analyses using the inverse variance method to assess whether conclusions change under the assumption of a single true effect. Substantial differences between random-effects and fixed-effect results would suggest important heterogeneity. For outcomes where meta-analysis is feasible, we will pool effect estimates and present results in forest plots showing individual study effects, pooled estimates with diamond markers, confidence intervals, heterogeneity statistics, and identification of subgroups when applicable. We will report both the random-effects and fixed-effect pooled estimates in Supplementary materials with the random-effects estimate as primary.

For multi-arm trials that compare multiple ERAS variants or intensities to a single control group, we will handle unit of analysis issues by either combining the intervention arms into a single group when appropriate or by splitting the control group into two or more groups with proportionally smaller sample sizes to avoid double-counting participants in the meta-analysis. The approach chosen will depend on whether the intervention arms are sufficiently similar to be meaningfully combined. For cluster-randomized trials, if any are identified, we will extract the intra-cluster correlation coefficient and use it to adjust the sample size to an effective sample size, or if this information is not available, we will conduct sensitivity analyses using a range of plausible intra-cluster correlation coefficients.

### Subgroup analyses and sensitivity analyses

To explore potential effect modifiers and sources of heterogeneity, we will conduct pre-specified subgroup analyses when sufficient studies are available, defined as at least three studies per subgroup. Subgroup analyses will examine whether the effect of ERAS protocols differs according to the number of ERAS components implemented, comparing protocols with five or more components versus those with fewer than five components, based on the hypothesis that more comprehensive ERAS protocols may be more effective. We will examine surgical approach, comparing posterior-only procedures versus combined anterior–posterior procedures, as combined procedures may present different challenges and opportunities for ERAS implementation. Power estimates for key subgroup analyses were informed by available published data. For the comparison of adolescent versus adult patients, we anticipate a minimum of 15 eligible studies per subgroup based on our preliminary search, yielding adequate power (≥80%) to detect a standardized mean difference of ≥0.5 in primary outcomes at *α* = 0.05. For the neuromuscular scoliosis subgroup, given the expected paucity of eligible studies (estimated n < 10), this analysis is designated as exploratory. Findings will be interpreted with caution and presented with wide confidence intervals. We will report the number of studies and total participants within each subgroup and will conduct sensitivity analyses to assess robustness. For each subgroup analysis, we will test for statistical interaction using the Chi-squared test for subgroup differences with *p* < 0.10 considered statistically significant given the low power of interaction tests. We will interpret subgroup findings cautiously, recognizing that subgroup analyses are observational even when based on randomized trials, are susceptible to false positives when multiple subgroups are examined, and should be hypothesis-generating rather than definitive. When ten or more studies are available for a given outcome, we will additionally conduct meta-regression analyses to examine whether effect sizes are associated with continuous study-level characteristics such as year of publication, mean patient age, mean number of levels fused, or mean baseline severity. Meta-regression will be performed using Stata version 17 with restricted maximum likelihood estimation. Given the risk of ecological fallacy and overfitting, meta-regression will be considered exploratory, and we will limit the number of covariates examined to maintain adequate degrees of freedom. To assess the robustness of our findings, we will conduct several sensitivity analyses. We will repeat meta-analyses excluding studies at high risk of bias to determine whether conclusions change when restricted to higher-quality evidence. We will exclude studies with imputed or digitally extracted data to evaluate the impact of data quality. We will compare random-effects versus fixed-effect models to assess the influence of statistical model assumptions. For continuous outcomes, we will convert all effect measures to the same scale and repeat analyses to ensure that choice of effect measure does not substantially alter conclusions. We will exclude studies with extreme effect sizes or high influence as identified through influence analyses to assess whether any single study disproportionately affects the pooled estimate. Results of sensitivity analyses will be presented in appendices, and we will discuss any instances where conclusions differ from the primary analysis. If substantial variation in ERAS components is observed across studies, we will conduct a network meta-analysis (using netmeta package in R) to compare different ERAS bundles indirectly against standard care. Missing standard deviations will be handled via multiple imputation by chained equations (MICE), incorporating study-level covariates (sample size, mean values). Complete-case analysis and sensitivity analyses will be performed to assess robustness.

### Assessment of publication bias

If ten or more studies are available for a given outcome, we will assess publication bias and small-study effects using multiple methods. We will visually inspect funnel plots for asymmetry, which may suggest publication bias although other factors such as heterogeneity or methodological differences can also cause asymmetry. We will conduct Egger’s regression test for continuous outcomes and Harbord’s test for dichotomous outcomes, with *p* < 0.10 considered statistically significant evidence of asymmetry. If evidence of publication bias is detected, we will use the trim-and-fill method to estimate the number of potentially missing studies and the adjusted effect size accounting for these missing studies. We will also examine whether smaller studies report systematically different effects than larger studies, which may indicate publication bias or genuine differences in effect by study size. We will acknowledge that with fewer than ten studies, power to detect publication bias is very limited, and absence of detected bias does not ensure absence of bias. We will also search trial registries to identify registered studies that have not been published and will note any identified studies with completed enrollment but no published results.

### Certainty of evidence assessment

The certainty of evidence for all outcomes will be assessed using the GRADE approach, which considers five domains that may decrease certainty: risk of bias, inconsistency, indirectness, imprecision, and publication bias, as well as three factors that may increase certainty: large magnitude of effect, dose–response gradient, and when all plausible confounding would reduce the demonstrated effect. Evidence from randomized controlled trials begins at high certainty and may be rated down, while evidence from observational studies begins at low certainty but may be rated up. For each outcome, we will provide a transparent rationale for rating decisions. Risk of bias will be assessed based on the overall risk of bias judgments from the RoB 2 or ROBINS-I assessments, with serious risk of bias leading to downgrading by one level and very serious risk leading to downgrading by two levels. Inconsistency will be evaluated based on statistical heterogeneity, with unexplained substantial heterogeneity suggesting downgrading. Indirectness will be considered if there are concerns about the applicability of evidence to our review question regarding populations, interventions, comparators, or outcomes. Imprecision will be assessed based on sample size, number of events, and width of confidence intervals, with downgrading when optimal information size is not met or confidence intervals include both clinically important benefit and harm. Publication bias will be evaluated using the methods described previously. The overall certainty for each outcome will be rated as high, indicating high confidence that the true effect lies close to the estimate; moderate, suggesting moderate confidence that the true effect is likely close to the estimate but possibly substantially different; low, meaning limited confidence with the true effect possibly substantially different; or very low, suggesting very little confidence with the true effect likely substantially different from the estimate. GRADE evidence profiles will be created for all outcomes showing the number of studies and participants, effect sizes with confidence intervals, heterogeneity statistics, and detailed explanations for all rating decisions. Summary of Findings tables will present the key results for primary and most important secondary outcomes in a format accessible to clinical and policy audiences.

### Additional analyses

If adequate data are available, we will conduct post-hoc exploratory analyses examining individual ERAS components to identify which elements appear most strongly associated with beneficial outcomes. This component-level analysis will be hypothesis-generating and interpreted with appropriate caution given potential confounding between components. We will also narratively synthesize information on implementation factors including barriers and facilitators to ERAS protocol adherence, staff training requirements, resource requirements, and contextual factors that may influence success of ERAS implementation, drawing on both quantitative process data and any qualitative data reported in included studies. If sufficient studies report cost data, we will attempt to standardize costs across studies and settings, converting all costs to a common currency and adjusting for inflation to a reference year, and will assess cost-effectiveness where feasible. However, we anticipate substantial heterogeneity in cost measurement and reporting that may limit quantitative synthesis.

### Execution criteria for advanced analyses

To avoid post-hoc data-driven conclusions and ensure methodological transparency, we pre-specify the following minimum study thresholds for advanced analyses. Subgroup analyses will only be performed when at least three studies are available per subgroup. If this threshold is not met, findings will be presented narratively instead of quantitative pooling. Meta-regression will only be conducted when at least ten studies are available for a given outcome. If fewer studies are available, we will only perform categorical subgroup analyses and omit meta-regression. Network meta-analysis will only be performed when at least three distinct ERAS bundle patterns can be identified with at least three studies per bundle. If this threshold is not met, we will conduct a narrative component-level analysis instead of network meta-analysis. Multiple imputation by chained equations (MICE) will only be applied when the proportion of studies with missing data is at least 30% and at least 15 studies are included. Otherwise, complete-case analysis will be used as the primary approach with sensitivity analyses to assess the impact of missing data. Core analyses (primary outcome, core secondary outcomes, basic sensitivity analyses, risk of bias assessment, GRADE evidence certainty grading) are prioritized and will be performed regardless of the number of eligible studies. All advanced analyses are designated as hypothesis-generating and are conditional on data availability.

### Equity considerations

In accordance with recommendations for equity-focused systematic reviews, we will apply the PROGRESS-Plus framework to assess how findings may differ across subpopulations defined by: Place of residence (high-income vs. low−/middle-income countries), Race/ethnicity/culture, Occupation, Gender/sex, Religion, Education, Socioeconomic status, and Social capital. Additional ‘Plus’ factors specific to our review include age group (pediatric vs. adult), scoliosis etiology (idiopathic vs. neuromuscular vs. congenital), and hospital resource setting (tertiary referral vs. community hospital). We will report PROGRESS-Plus stratification where data are available and will explicitly discuss gaps in equity-relevant reporting in the Limitations section of our forthcoming results paper.

### Ethics and dissemination

This systematic review will synthesize data from published studies and will not involve collection of individual patient data or direct patient contact. Therefore, ethical approval is not required. All data extraction and analysis will be conducted in accordance with standard systematic review methodology and reported following international guidelines.

The findings of this systematic review will be disseminated through multiple channels to reach diverse stakeholder audiences. We will submit the completed systematic review for publication in a peer-reviewed journal, preferably an open-access journal to maximize accessibility to clinicians, researchers, patients, and policymakers. The manuscript will follow the PRISMA 2020 reporting guidelines and will include all required Supplementary materials. The review protocol, search strategies, data extraction forms, and complete dataset will be made available as Supplementary materials or upon reasonable request to support transparency and enable replication or extension of our work. Any amendments to this protocol will be documented in detail including the date of amendment, the specific change made, and the rationale for the change. All amendments will be reported in the final manuscript to maintain transparency. If substantive changes are made that affect the review question or methods, we will update our PROSPERO registration accordingly.

## Discussion

ERAS protocols represent a paradigm shift in perioperative management, emphasizing the integration of evidence-based interventions throughout the surgical pathway to optimize patient recovery. In scoliosis correction surgery, which is often associated with significant morbidity and prolonged convalescence, the adaptation of ERAS principles offers the potential to address long-standing challenges related to pain control, mobilization, and healthcare resource utilization. Despite the increasing adoption of ERAS pathways in various surgical specialties, their role in scoliosis correction surgery has not been comprehensively defined (8). Previous studies in spine surgery populations have demonstrated that ERAS protocols may reduce the length of hospital stay, minimize postoperative complications, and decrease opioid requirements. However, the majority of available evidence comes from heterogeneous patient groups or procedures distinct from scoliosis correction, limiting the generalizability of these findings to this specific population.

### Strengths of this review

This systematic review and meta-analysis protocol addresses these limitations by focusing exclusively on studies comparing ERAS protocols with conventional care in patients undergoing scoliosis correction surgery. By emphasizing methodological rigor and clear inclusion criteria, this review aims to generate robust and clinically relevant conclusions regarding the true impact of ERAS implementation in this field. The review will evaluate a broad spectrum of outcomes, including not only length of stay and complication rates but also patient-centered measures such as pain scores, satisfaction, functional recovery, and economic outcomes. Such comprehensive assessment recognizes that perioperative success extends beyond traditional clinical endpoints to encompass the quality of the patient’s recovery experience and the sustainability of healthcare delivery. Our protocol demonstrates several methodological strengths. We will conduct a comprehensive search across multiple databases without language restrictions, reducing the risk of missing relevant evidence. The use of validated, contemporary risk of bias assessment tools appropriate to study design, including RoB 2 for randomized trials and ROBINS-I for non-randomized studies, will enable rigorous quality appraisal (21). Independent duplicate screening, data extraction, and quality assessment with clear procedures for resolving disagreements will minimize errors and bias in the review process. Pre-specified subgroup and sensitivity analyses will allow exploration of heterogeneity and assessment of robustness of findings. The application of the GRADE approach to assess evidence certainty will enhance the transparency and credibility of our conclusions (22, 23). The GRADE framework provides a systematic and transparent process for rating the certainty of evidence across outcomes, considering factors such as risk of bias, inconsistency, indirectness, imprecision, and publication bias (24). Comprehensive reporting following PRISMA guidelines and public registration in PROSPERO will ensure transparency and enable scrutiny of our methods (25).

### Anticipated challenges and limitations

Several anticipated challenges may influence the synthesis and interpretation of evidence. There may be substantial variability in the composition and intensity of ERAS protocols among studies, with some studies implementing comprehensive protocols including ten or more elements while others may include only three or four core components (12). This heterogeneity has been observed across different surgical specialties implementing ERAS, and spine surgery is no exception. Additionally, there may be variation in the definition of conventional care, with some control groups receiving care that includes certain ERAS elements implemented in an unstructured manner. This heterogeneity in both intervention and comparator could affect both the magnitude and consistency of observed effects. The inclusion of patients across a wide age range from adolescents aged 10 years to elderly adults, and with different scoliosis etiologies ranging from idiopathic to neuromuscular and degenerative types, introduces additional complexity (15). These different patient populations may respond differently to ERAS protocols due to varying physiological reserves, comorbidity burdens, and baseline functional status, necessitating planned subgroup analyses to explore potential effect modifiers. Patient-specific factors including age, body mass index, and comorbidities have been shown to significantly influence perioperative outcomes and healthcare resource utilization in spine surgery (2). Variations in outcome reporting and follow-up durations across studies could limit the ability to perform quantitative synthesis for some endpoints. Some studies may report length of stay in categorical terms rather than as continuous data, or may report pain at different time points than other studies, complicating pooling efforts. Additionally, definitions of complications may vary, with some studies providing detailed classification while others report only overall complication rates. The majority of available evidence may come from observational studies rather than randomized controlled trials, given the challenges of randomizing complex multimodal interventions and the pragmatic nature of much ERAS implementation research. This will necessitate careful consideration of potential confounding and bias in interpreting results, and we will rely heavily on the ROBINS-I tool to assess these risks systematically. Publication bias and selective reporting may affect the available evidence, with positive studies more likely to be published than those showing no benefit. We will assess this systematically using funnel plots and statistical tests when sufficient studies are available, and will search trial registries to identify completed but unpublished studies. Generalizability of findings to diverse healthcare settings may be uncertain, as most studies are likely to come from high-income countries with well-resourced healthcare systems. Implementation of comprehensive ERAS protocols requires infrastructure, multidisciplinary coordination, and resources that may not be available in all settings, potentially limiting the external validity of pooled results. Studies have shown that compliance with ERAS protocol components can be highly variable, and successful implementation often requires multidisciplinary teams including surgeons, anesthesiologists, nurses, physiotherapists, and other specialty personnel (9). Given the wide range of patient ages and diverse scoliosis etiologies included, the overall pooled effect size should be interpreted with caution as a general reference. Subgroup stratified results by age group, etiology and surgical approach will provide more clinically actionable information for specific patient populations, and will be the focus of result interpretation. Furthermore, if substantial clinical heterogeneity is identified, we will prioritize reporting subgroup results and will not force an overall pooled estimate if it lacks clinical interpretability.

### Mitigation strategies

To mitigate these challenges, this review will employ several strategies. We will conduct detailed extraction of ERAS protocol elements and conventional care practices to enable clear description of what interventions were actually compared. Pre-specified subgroup analyses by number of ERAS components, patient age, surgical approach, and study design will help explore sources of heterogeneity and identify circumstances under which ERAS may be most beneficial. Sensitivity analyses excluding high risk of bias studies and those with imputed data will assess the robustness of findings to methodological limitations. The GRADE approach will provide transparent assessment of evidence certainty, allowing users to understand the confidence warranted by the evidence. Narrative synthesis will be provided alongside meta-analysis to capture nuances that quantitative synthesis may obscure, including implementation factors and contextual considerations.

### Clinical and research implications

Ultimately, the results of this review are expected to inform clinical practice by clarifying the efficacy and safety of ERAS pathways in scoliosis correction surgery. Should ERAS protocols demonstrate consistent benefit across outcomes with acceptable safety profiles, these findings may support broader adoption and standardization of ERAS programs for scoliosis patients, leading to improvements in recovery trajectories, patient experience, and resource allocation. Hospitals and surgical teams considering ERAS implementation will have synthesized evidence to guide decision-making and protocol development. The review may also identify which specific ERAS components or combinations of components appear most strongly associated with benefit, although these findings will be hypothesis-generating and require confirmation in future trials specifically designed to evaluate individual components. This could inform efforts to develop streamlined, efficient ERAS protocols that focus on the most impactful interventions. Conversely, if evidence of benefit is limited, inconsistent, or accompanied by concerning safety signals, this would underscore the need for further high-quality research before widespread adoption. The review may identify specific gaps in the evidence base, such as lack of long-term outcome data, insufficient evaluation in particular patient subgroups, or absence of rigorous cost-effectiveness analyses, that should be priorities for future investigation. Economic considerations are particularly important given the substantial costs associated with spine surgery and the need for value-based care. Cost-effectiveness analyses in spine surgery have shown considerable variability in methodology and reporting, highlighting the need for standardized approaches to economic evaluation. While some studies have demonstrated that ERAS protocols can reduce healthcare costs through decreased length of stay and fewer complications, others have found more nuanced results when considering implementation costs. Future economic evaluations should adopt comprehensive perspectives, including both healthcare system and societal costs, and should utilize standardized methodologies to better understand the true economic impact of ERAS implementation. Future research recommendations emerging from this review may include calls for adequately powered, multi-center randomized controlled trials with standardized ERAS protocols and clearly defined conventional care comparators; longer-term follow-up to assess whether perioperative benefits translate into sustained improvements in quality of life and functional outcomes; evaluation of patient-reported outcomes and patient preferences regarding ERAS pathway elements; detailed economic analyses including cost-effectiveness and budget impact assessments; implementation science research examining facilitators and barriers to ERAS adoption and strategies for successful implementation across diverse settings; and investigation of specific ERAS components through factorial designs or adaptive trial approaches to identify optimal protocol configurations.

## Conclusion

In summary, this systematic review and meta-analysis will fill a critical knowledge gap in perioperative care for scoliosis correction surgery and provide actionable insights for clinicians, policymakers, and researchers committed to advancing surgical recovery and patient outcomes. By rigorously synthesizing the best available evidence, critically appraising methodological quality, and transparently rating certainty of evidence, this review will enable evidence-informed decision-making regarding ERAS implementation for scoliosis surgery patients. The findings will contribute to the growing body of literature on ERAS in spinal surgery and may catalyze further research and quality improvement efforts in this important area of perioperative care.

## Data Availability

The datasets used and analyzed during the current study are available from the corresponding author on reasonable request.
