# Supplementary material for: Enhanced recovery after surgery in patients undergoing scoliosis correction surgery: protocol for a systematic review and meta-analysis
Source: Front Med (Lausanne). 2026 Jul 28;13:1872301. doi: 10.3389/fmed.2026.1872301 (PMC13457733; doi:10.3389/fmed.2026.1872301)
Supplement: Supplementary file 1 [file Table_1.DOCX]

**Supplementary File 1 PRISMA-P (Preferred Reporting Items for Systematic review and Meta-Analysis Protocols)**

| Section and topic | Item No | Checklist item | page |
| --- | --- | --- | --- |
| ADMINISTRATIVE INFORMATION | | |  |
| Title: |  |  |  |
| Identification | 1a | Identify the report as a protocol of a systematic review | p1 |
| Update | 1b | If the protocol is for an update of a previous systematic review, identify as such | not applicable |
| Registration | 2 | If registered, provide the name of the registry (such as PROSPERO) and registration number | p2+p4 |
| Authors: |  |  |  |
| Contact | 3a | Provide name, institutional affiliation, e-mail address of all protocol authors; provide physical mailing address of corresponding author | p1 |
| Contributions | 3b | Describe contributions of protocol authors and identify the guarantor of the review | p1 |
| Amendments | 4 | If the protocol represents an amendment of a previously completed or published protocol, identify as such and list changes; otherwise, state plan for documenting important protocol amendments | p5 |
| Support: |  |  |  |
| Sources | 5a | Indicate sources of financial or other support for the review | p1 |
| Sponsor | 5b | Provide name for the review funder and/or sponsor | p1 |
| Role of sponsor or funder | 5c | Describe roles of funder(s), sponsor(s), and/or institution(s), if any, in developing the protocol | not applicable |
| INTRODUCTION | | |  |
| Rationale | 6 | Describe the rationale for the review in the context of what is already known | p3–p4 |
| Objectives | 7 | Provide an explicit statement of the question(s) the review will address with reference to participants, interventions, comparators, and outcomes (PICO) | P4 |
| METHODS | | |  |
| Eligibility criteria | 8 | Specify the study characteristics (such as PICO, study design, setting, time frame) and report characteristics (such as years considered, language, publication status) to be used as criteria for eligibility for the review | P4–p7 |
| Information sources | 9 | Describe all intended information sources (such as electronic databases, contact with study authors, trial registers or other grey literature sources) with planned dates of coverage | p7 |
| Search strategy | 10 | Present draft of search strategy to be used for at least one electronic database, including planned limits, such that it could be repeated | p7-p8 |
| Study records: |  |  |  |
| Data management | 11a | Describe the mechanism(s) that will be used to manage records and data throughout the review | p8 |
| Selection process | 11b | State the process that will be used for selecting studies (such as two independent reviewers) through each phase of the review (that is, screening, eligibility and inclusion in meta-analysis) | p8-p9 |
| Data collection process | 11c | Describe planned method of extracting data from reports (such as piloting forms, done independently, in duplicate), any processes for obtaining and confirming data from investigators | P9–p10 |
| Data items | 12 | List and define all variables for which data will be sought (such as PICO items, funding sources), any pre-planned data assumptions and simplifications | p9–p10 |
| Outcomes and prioritization | 13 | List and define all outcomes for which data will be sought, including prioritization of main and additional outcomes, with rationale | p6 |
| Risk of bias in individual studies | 14 | Describe anticipated methods for assessing risk of bias of individual studies, including whether this will be done at the outcome or study level, or both; state how this information will be used in data synthesis | p10–p11 |
| Data synthesis | 15a | Describe criteria under which study data will be quantitatively synthesised | p11-p12 |
|  | 15b | If data are appropriate for quantitative synthesis, describe planned summary measures, methods of handling data and methods of combining data from studies, including any planned exploration of consistency (such as I^2^, Kendall’s τ) | p11-p13 |
|  | 15c | Describe any proposed additional analyses (such as sensitivity or subgroup analyses, meta-regression) | p13-p14 |
|  | 15d | If quantitative synthesis is not appropriate, describe the type of summary planned | p12 |
| Meta-bias(es) | 16 | Specify any planned assessment of meta-bias(es) (such as publication bias across studies, selective reporting within studies) | p14 |
| Confidence in cumulative evidence | 17 | Describe how the strength of the body of evidence will be assessed (such as GRADE) | p14–p15 |

*** It is strongly recommended that this checklist be read in conjunction with the PRISMA-P Explanation and Elaboration (cite when available) for important clarification on the items. Amendments to a review protocol should be tracked and dated. The copyright for PRISMA-P (including checklist) is held by the PRISMA-P Group and is distributed under a Creative Commons Attribution Licence 4.0.**

*From: Shamseer L, Moher D, Clarke M, Ghersi D, Liberati A, Petticrew M, Shekelle P, Stewart L, PRISMA-P Group. Preferred reporting items for systematic review and meta-analysis protocols (PRISMA-P) 2015: elaboration and explanation. BMJ. 2015 Jan 2;349(jan02 1):g7647.*

**Supplementary File 2 – ERAS Components for Scoliosis Correction Surgery**

The following table lists the multimodal ERAS components used for eligibility assessment and data extraction. Studies must implement a minimum of three components spanning at least two perioperative phases for inclusion.

| **Phase** | **ERAS Component** | **Examples** | **Evidence Basis** |
| --- | --- | --- | --- |
| **Preoperative** | Patient education and counseling | Written/verbal information about surgery, expected recovery, and discharge goals | ERAS Society Guidelines; Ljungqvist 2017 |
| **Preoperative** | Nutritional assessment and optimization | Carbohydrate loading (≤2h before surgery); nutritional supplementation for malnourished patients | Smith 2014; Weimann 2017 |
| **Preoperative** | Prehabilitation | Preoperative physiotherapy, respiratory exercises, core strengthening | Scheede-Bergdahl 2019 |
| **Preoperative** | Optimization of comorbidities | Anemia correction (iron therapy, EPO); smoking cessation ≥4 weeks prior | Muñoz 2017 |
| **Preoperative** | Avoidance of prolonged fasting | Clear fluids until 2h; solids until 6h before anesthesia | ASA Guidelines 2017 |
| **Intraoperative** | Multimodal analgesia / opioid-sparing strategy | Neuraxial blocks, intrathecal morphine, ketamine, NSAIDs, acetaminophen | Dunn 2016; Garg 2020 |
| **Intraoperative** | Tranexamic acid (TXA) administration | IV or topical TXA to reduce blood loss | Verma 2014; Li 2017 |
| **Intraoperative** | Goal-directed fluid therapy (GDFT) | Intraoperative hemodynamic monitoring; stroke volume variation-guided fluid management | Hamilton 2011 |
| **Intraoperative** | Maintenance of normothermia | Active warming (forced-air blanket); IV fluid warming; ambient temperature control | Scott 2015 |
| **Intraoperative** | Minimally invasive or reduced-invasiveness techniques | MIS approaches; reduced retraction time; neuromonitoring | Phan 2017 |
| **Postoperative** | Multimodal pain management | Scheduled acetaminophen + NSAIDs + gabapentinoids; patient-controlled analgesia if needed | Garg 2020; Dietz 2020 |
| **Postoperative** | Early mobilization | Standing/walking within 24h of surgery; structured physiotherapy protocol | ERAS Society 2019 |
| **Postoperative** | Early enteral nutrition | Oral intake resumed within 4–6h postoperatively; dietitian review | Ljungqvist 2017 |
| **Postoperative** | Avoidance of routine drains and catheters | Urinary catheter removal ≤24h; surgical drain removal per protocol | ERAS Society 2019 |
| **Postoperative** | Venous thromboembolism (VTE) prophylaxis | LMWH + mechanical compression stockings; early ambulation | Bauer 2010 |
| **Postoperative** | Nausea and vomiting (PONV) prophylaxis | Dexamethasone + ondansetron; avoidance of N₂O | Gan 2020 |
| **Postoperative** | Structured discharge planning | Discharge criteria checklist; patient education on warning signs; follow-up appointment | ERAS Society 2019 |

Note: Studies must incorporate a minimum of THREE of the above components across at least TWO perioperative phases (preoperative, intraoperative, postoperative) to qualify for inclusion. This definition is adapted from the ERAS Society Guidelines for Spine Surgery and relevant published systematic reviews.

**References for Table 1 – ERAS Components (Vancouver Format)**

*The following references correspond to the Evidence Basis column of Table 1. Numbers in brackets [n] within the table correspond to the entries below.*

**Preoperative Phase**

**1.** Ljungqvist O, Scott M, Fearon KC. Enhanced Recovery After Surgery: A Review. JAMA Surg. 2017;152(3):292-298. doi:10.1001/jamasurg.2016.4952

**2.** Davies B, Yee A, Batke J, Dietz N, Dea N, Street J. Consensus statement for perioperative care in lumbar spinal fusion: Enhanced Recovery After Surgery (ERAS®) Society recommendations. Spine J. 2021;21(5):729-752. doi:10.1016/j.spinee.2021.01.001

**3.** Weimann A, Braga M, Carli F, Higashiguchi T, Hübner M, Klek S, et al. ESPEN guideline: Clinical nutrition in surgery. Clin Nutr. 2017;36(3):623-650. doi:10.1016/j.clnu.2017.02.013

**4.** Scheede-Bergdahl C, Minnella EM, Carli F. Multi-modal prehabilitation: addressing the why, when, what, how, who and where next. Anaesthesia. 2019;74(Suppl 1):20-26. doi:10.1111/anae.14505

**5.** Muñoz M, Acheson AG, Auerbach M, Besser M, Habler O, Kehlet H, et al. International consensus statement on the peri-operative management of anaemia and iron deficiency. Anaesthesia. 2017;72(2):233-247. doi:10.1111/anae.13773

**6.** Practice Guidelines for Preoperative Fasting and the Use of Pharmacologic Agents to Reduce the Risk of Pulmonary Aspiration: An Updated Report by the American Society of Anesthesiologists Task Force. Anesthesiology. 2017;126(3):376-393. doi:10.1097/ALN.0000000000001452

**Intraoperative Phase**

**7.** Garg B, Dixit V, Batra S, Mehta N. Enhanced recovery after surgery in spinal surgery: a review of the literature. J Clin Orthop Trauma. 2020;11(5):744-749. doi:10.1016/j.jcot.2020.06.025

**8.** Dietz N, Sharma M, Adams S, Alhourani A, Ugiliweneza B, Wang D, et al. Enhanced Recovery After Surgery (ERAS) for Spine Surgery: A Systematic Review. World Neurosurg. 2020;140:614-626. doi:10.1016/j.wneu.2020.04.143

**9.** Verma K, Errico T, Diefenbach C, Hoelscher C, Peters A, Huncke T, et al. The relative efficacy of antifibrinolytics in adolescent idiopathic scoliosis: a prospective randomized trial. J Bone Joint Surg Am. 2014;96(10):e80. doi:10.2106/JBJS.L.00008

**10.** Li G, Sun TW, Luo G, Zhang C. Efficacy of antifibrinolytic agents on surgical bleeding and transfusion requirements in spine surgery: a meta-analysis. Eur Spine J. 2017;26(1):140-154. doi:10.1007/s00586-016-4792-x

**11.** Hamilton MA, Cecconi M, Rhodes A. A systematic review and meta-analysis on the use of preemptive hemodynamic intervention to improve postoperative outcomes in moderate and high-risk surgical patients. Anesth Analg. 2011;112(6):1392-1402. doi:10.1213/ANE.0b013e3181eeaae5

**12.** Scott AV, Stonemetz JL, Wasey JO, Johnson DJ, Rivers RJ, Koch CG, et al. Compliance with Surgical Care Improvement Project for Body Temperature Management (SCIP Inf-10) Is Associated with Improved Clinical Outcomes. Anesthesiology. 2015;123(1):116-125. doi:10.1097/ALN.0000000000000681

**13.** Phan K, Rao PJ, Kam AC, Mobbs RJ. Minimally invasive versus open transforaminal lumbar interbody fusion for treatment of degenerative lumbar disease: systematic review and meta-analysis. Eur Spine J. 2015;24(5):1017-1030. doi:10.1007/s00586-015-3903-4

**Postoperative Phase**

**14.** Gadiya AD, Koch JEJ, Yoon WW, Demetriades AK. Enhanced recovery after surgery (ERAS) in adolescent idiopathic scoliosis (AIS): a meta-analysis and systematic review. Spine Deform. 2021;9(4):893-904. doi:10.1007/s43390-021-00310-w

**15.** Gan TJ, Belani KG, Bergese S, Chung F, Diemunsch P, Habib AS, et al. Fourth Consensus Guidelines for the Management of Postoperative Nausea and Vomiting. Anesth Analg. 2020;131(2):411-448. doi:10.1213/ANE.0000000000004833

**16.** Bauer KA. Pros and cons of new oral anticoagulants. Hematology Am Soc Hematol Educ Program. 2013;2013:464-470. doi:10.1182/asheducation-2013.1.464

**17.** Zheng F, Cammisa FP Jr, Sandhu HS, Girardi FP, Khan SN. Factors predicting hospital stay, operative time, blood loss, and transfusion in patients undergoing revision posterior lumbar spine decompression, fusion, and segmental instrumentation. Spine. 2002;27(8):818-824. doi:10.1097/00007632-200204150-00008

** References 1 and 2 are cited across multiple ERAS components. Reference 1 (Ljungqvist 2017) serves as the foundational ERAS overview; Reference 2 (Davies 2021) represents the ERAS® Society Guidelines specifically for lumbar spinal fusion and is the primary evidence source for this review.*
